# Subproteomic profiling of sarcolemma from dystrophic *mdx-4cv* skeletal muscle

**DOI:** 10.1016/j.dib.2018.02.020

**Published:** 2018-02-16

**Authors:** Sandra Murphy, Margit Zweyer, Michael Henry, Paula Meleady, Rustam R. Mundegar, Dieter Swandulla, Kay Ohlendieck

**Affiliations:** a Department of Biology, Maynooth University, National University of Ireland, Maynooth, Co. Kildare, Ireland; b Institute of Physiology II, University of Bonn, D-53115 Bonn, Germany; c National Institute for Cellular Biotechnology, Dublin City University, Dublin 9, Ireland

## Abstract

The proteomic data presented in this article provide supporting information to the related research article "Proteomic analysis of the sarcolemma-enriched fraction from dystrophic *mdx-4cv* skeletal muscle" (Murphy et al., 2018) [1]. In the associated research article, the sarcolemma from normal versus dystrophic skeletal muscle was analyzed by mass spectrometry-based proteomics. Sarcolemma vesicles were enriched by a lectin agglutination method and then analyzed by liquid chromatography tandem mass spectrometry. Here we provide additional datasets on proteins with decreased versus increased abundance in dystrophin-deficient muscle plasma membranes.

**Specifications table**

**Table:** 

Subject area	*Biology*
More specific subject area	*Biomedicine*
Type of data	*Tables*
How data was acquired	*LC-MS/MS*
Data format	*Analyzed*
Experimental factors	*Protein was extracted from sarcolemma fraction from wild type versus dystrophic mdx-4cv skeletal muscle specimens.*
Experimental features	*Comparative mass spectrometry-based proteomic profiling of the sarcolemma-enriched fraction that has been isolated by lectin affinity agglutination.*
Data source location	*Maynooth, Ireland*
Data accessibility	*The data are available with this article*

**Value of the data**

• Proteomic data presented here provide an overview of protein changes in X-linked muscular dystrophy.
• This data provides additional listings of protein alterations in the sarcolemma-enriched fraction from dystrophic skeletal muscle tissue.
• The mass spectrometric data are valuable to serve as a pathobiochemical signature of dystrophin-deficient muscle fibres.

## 1 Data

The data presented relate to the systematic survey of dystrophic skeletal muscle tissue using mass spectrometry-based proteomics of the sarcolemma from the *mdx-4cv* mouse model of Duchenne muscular dystrophy [1]. Table 1 lists the mass spectrometric identification of proteins with a 1.6- to 4.9-fold increase in the sarcolemma-enriched fraction from dystrophic skeletal muscle. Listed are the accession number, the gene name, the protein name, the number of unique peptides, the peptide count, the confidence score, Anova (p) values and the maximum fold change of identified protein species. Table 2, Table 3 list the mass spectrometric identification of proteins identified by a single unique peptide with a reduced or increased abundance in the sarcolemma-enriched fraction from dystrophic *mdx-4cv* skeletal muscle, respectively.

**Table 1:** Mass spectrometric identification of proteins with a 1.6- to 4.9-fold increase in the sarcolemma-enriched fraction from dystrophic *mdx-4cv* skeletal muscle.

**Accession**	**Gene name**	**Protein name**	**Unique peptides**	**Peptide count**	**Confidence score**	**Anova (p)**	**Max fold change**
O08917	Flot1	Flotillin-1	3	3	191.2	2.01E-03	4.9
Q61738	Itga7	Integrin alpha-7	3	3	102.6	4.70E-05	4.8
P12970	Rpl7a	60S ribosomal protein L7a	2	2	88.3	9.78E-08	4.8
P27773	Pdia3	Protein disulfide-isomerase A3	4	4	122.0	5.40E-05	4.8
Q9DBS1	Tmem43	Transmembrane protein 43	3	3	97.0	2.21E-08	4.7
P08226	Apoe	Apolipoprotein E	5	5	200.7	2.40E-05	4.6
Q8CC88	Vwa8	von Willebrand factor A domain-containing protein 8	2	2	50.7	2.81E-03	4.6
Q8VDD5	Myh9	Myosin-9	3	3	119.6	2.12E-06	4.6
P70302	Stim1	Stromal interaction molecule 1	2	2	3.2	3.28E-05	4.6
P13595	Ncam1	Neural cell adhesion molecule 1	3	3	71.5	1.57E-05	4.5
Q9EQK5	Mvp	Major vault protein	2	2	57.3	7.84E-06	4.5
Q60634	Flot2	Flotillin-2	4	4	267.1	3.60E-07	4.4
Q61830	Mrc1	Macrophage mannose receptor 1	12	12	491.7	5.40E-04	4.4
Q3U7R1	Esyt1	Extended synaptotagmin-1	3	3	51.3	1.35E-05	4.3
Q9DCN2	Cyb5r3	NADH-cytochrome b5 reductase 3	2	2	103.9	4.05E-07	4.3
Q07113	Igf2r	Cation-independent mannose-6-phosphate receptor	3	3	50.2	8.66E-05	4.2
A2ASS6	Ttn	Titin	105	105	3529.1	9.91E-04	4.2
Q9DC69	Ndufa9	NADH dehydrogenase [ubiquinone] 1 alpha subcomplex subunit 9, mitochondrial	2	2	141.7	7.94E-04	4.0
Q5XKE0	Mybpc2	Myosin-binding protein C, fast-type	3	3	239.0	5.42E-04	4.0
P14131	Rps16	40S ribosomal protein S16	3	3	137.6	3.60E-07	3.9
P19324	Serpinh1	Serpin H1	3	3	80.5	2.01E-05	3.9
Q8BY89	Slc44a2	Choline transporter-like protein 2	2	2	54.5	7.41E-04	3.8
P47740	Aldh3a2	Fatty aldehyde dehydrogenase	2	2	101.6	2.89E-05	3.8
P22315	Fech	Ferrochelatase, mitochondrial	2	2	3.6	2.79E-03	3.8
P80318	Cct3	T-complex protein 1 subunit gamma	2	2	65.6	4.94E-05	3.7
Q04857	Col6a1	Collagen alpha-1(VI) chain	2	2	5.4	2.88E-05	3.7
Q61941	Nnt	NAD(P) transhydrogenase, mitochondrial	2	2	63.0	1.25E-03	3.7
Q62159	Rhoc	Rho-related GTP-binding protein RhoC	2	2	61.0	1.22E-08	3.7
P35980	Rpl18	60S ribosomal protein L18	2	2	53.0	3.20E-03	3.6
P09055	Itgb1	Integrin beta-1	3	3	5.4	2.28E-04	3.6
P25444	Rps2	40S ribosomal protein S2	3	3	108.8	4.15E-06	3.6
Q91ZX7	Lrp1	Prolow-density lipoprotein receptor-related protein 1	4	4	184.3	4.03E-04	3.5
P48678	Lmna	Prelamin-A/C	5	5	166.1	1.51E-06	3.5
O55143	Atp2a2	Sarcoplasmic/endoplasmic reticulum calcium ATPase 2	7	7	370.1	1.16E-06	3.4
P51863	Atp6v0d1	V-type proton ATPase subunit d 1	3	3	116.1	6.71E-05	3.4
Q6ZWN5	Rps9	40S ribosomal protein S9	6	6	212.1	4.32E-08	3.4
Q8BFR5	Tufm	Elongation factor Tu, mitochondrial	2	2	4.4	2.80E-03	3.3
Q9JI91	Actn2	Alpha-actinin-2	7	7	251.5	1.86E-04	3.3
Q61475	Cd55	Complement decay-accelerating factor, GPI-anchored	2	2	83.8	5.51E-05	3.3
Q64133	Maoa	Amine oxidase [flavin-containing] A	4	4	196.2	8.22E-06	3.3
Q9JHU4	Dync1h1	Cytoplasmic dynein 1 heavy chain 1	2	2	53.6	2.27E-08	3.2
Q60936	Coq8a	Atypical kinase COQ8A, mitochondrial	2	2	117.5	1.61E-03	3.2
Q9D517	Agpat3	1-acyl-sn-glycerol-3-phosphate acyltransferase gamma	3	3	139.7	5.17E-05	3.2
Q8JZQ2	Afg3l2	AFG3-like protein 2	4	4	94.8	9.47E-06	3.1
Q9CXR1	Dhrs7	Dehydrogenase/reductase SDR family member 7	2	2	3.6	4.11E-04	3.1
O09161	Casq2	Calsequestrin-2	4	4	74.3	9.36E-04	3.1
Q68FD5	Cltc	Clathrin heavy chain 1	5	5	191.3	2.18E-04	3.0
Q02788	Col6a2	Collagen alpha-2(VI) chain	3	3	88.7	1.26E-06	3.0
P14206	Rpsa	40S ribosomal protein SA	2	2	135.0	4.63E-04	3.0
Q8BIJ6	Iars2	Isoleucine--tRNA ligase, mitochondrial	3	3	108.1	1.13E-04	3.0
Q9CZR2	Naalad2	N-acetylated-alpha-linked acidic dipeptidase 2	3	3	139.0	1.20E-06	3.0
P62852	Rps25	40S ribosomal protein S25	2	2	102.2	5.17E-04	3.0
P62270	Rps18	40S ribosomal protein S18	4	4	248.8	4.28E-04	3.0
Q9D0L4	Adck1	Uncharacterized aarF domain-containing protein kinase 1	2	2	102.9	2.46E-03	3.0
Q8VCM8	Ncln	Nicalin	2	2	51.2	2.21E-04	2.9
P62281	Rps11	40S ribosomal protein S11	2	2	97.3	2.85E-05	2.9
P97429	Anxa4	Annexin A4	2	2	94.3	4.42E-05	2.9
P42932	Cct8	T-complex protein 1 subunit theta	2	2	105.7	2.84E-05	2.9
Q61838	Pzp	Pregnancy zone protein	3	3	143.1	1.04E-04	2.8
Q9Z1E4	Gys1	Glycogen [starch] synthase, muscle	4	4	72.8	1.36E-06	2.8
Q91V79	Fitm1	Fat storage-inducing transmembrane protein 1	3	3	222.1	1.85E-04	2.8
Q9ET30	Tm9sf3	Transmembrane 9 superfamily member 3	2	2	51.3	1.94E-05	2.7
P62267	Rps23	40S ribosomal protein S23	2	2	124.9	2.66E-04	2.7
Q8C129	Lnpep	Leucyl-cystinyl aminopeptidase	2	2	90.9	8.18E-06	2.7
Q8BW75	Maob	Amine oxidase [flavin-containing] B	5	5	172.5	5.15E-07	2.7
P97351	Rps3a	40S ribosomal protein S3a	3	3	63.5	4.12E-06	2.6
Q8VDM4	Psmd2	26S proteasome non-ATPase regulatory subunit 2	2	2	113.6	1.94E-03	2.6
Q61543	Glg1	Golgi apparatus protein 1	2	2	57.8	3.74E-05	2.6
P47738	Aldh2	Aldehyde dehydrogenase, mitochondrial	2	2	69.3	2.25E-04	2.6
Q8CI59	Steap3	Metalloreductase STEAP3	3	3	137.0	6.01E-05	2.6
P10852	Slc3a2	4F2 cell-surface antigen heavy chain	2	2	56.8	6.48E-05	2.6
O70251	Eef1b	Elongation factor 1-beta	2	2	108.6	3.45E-03	2.6
P43406	Itgav	Integrin alpha-V	3	3	146.7	1.68E-04	2.6
P80317	Cct6a	T-complex protein 1 subunit zeta	2	2	104.9	2.08E-03	2.5
P13020	Gsn	Gelsolin	2	2	42.4	1.30E-03	2.5
Q811U4	Mfn1	Mitofusin-1	2	2	112.7	2.31E-06	2.5
P48036	Anxa5	Annexin A5	2	2	121.8	3.13E-03	2.5
P20152	Vim	Vimentin	4	4	141.9	1.82E-04	2.5
Q91WC9	Daglb	Sn1-specific diacylglycerol lipase beta	2	2	60.8	8.23E-03	2.4
Q8R127	Sccpdh	Saccharopine dehydrogenase-like oxidoreductase	3	3	119.6	4.02E-05	2.4
E9PZQ0	Ryr1	Ryanodine receptor 1	3	3	173.4	4.78E-03	2.4
P62242	Rps8	40S ribosomal protein S8	3	3	122.2	2.16E-04	2.4
Q61334	Bcap29	B-cell receptor-associated protein 29	3	3	54.6	6.40E-04	2.4
Q99P72	Rtn4	Reticulon-4	3	3	48.5	4.66E-07	2.3
Q921I1	Tf	Serotransferrin	2	2	97.0	6.81E-04	2.3
Q9WTR5	Cdh13	Cadherin-13	2	2	66.3	9.61E-04	2.3
Q9WUQ2	Preb	Prolactin regulatory element-binding protein	3	3	165.3	3.41E-05	2.3
Q9WV91	Ptgfrn	Prostaglandin F2 receptor negative regulator	2	2	118.6	1.62E-04	2.3
Q9D379	Ephx1	Epoxide hydrolase 1	3	3	52.5	4.69E-04	2.3
Q9Z2I0	Letm1	LETM1 and EF-hand domain-containing protein 1, mitochondrial	3	3	63.2	2.36E-03	2.2
Q924X2	Cpt1b	Carnitine O-palmitoyltransferase 1, muscle isoform	3	3	187.0	1.92E-03	2.2
Q8C7X2	Emc1	ER membrane protein complex subunit 1	3	3	132.6	8.08E-04	2.2
P14602	Hspb1	Heat shock protein beta-1	2	2	83.8	1.21E-05	2.2
P35293	Rab18	Ras-related protein Rab-18	3	3	74.2	1.55E-04	2.2
Q6PIE5	Atp1a2	Sodium/potassium-transporting ATPase subunit alpha-2	4	4	96.3	6.76E-04	2.1
O35114	Scarb2	Lysosome membrane protein 2	3	3	186.0	3.26E-04	2.1
Q9D6U8	Fam162a	Protein FAM162A	2	2	4.0	5.57E-04	2.1
Q9DBH5	Lman2	Vesicular integral-membrane protein VIP36	7	7	188.6	9.85E-06	2.1
Q05144	Rac2	Ras-related C3 botulinum toxin substrate 2	2	2	49.7	1.72E-04	2.1
Q91YQ5	Rpn1	Dolichyl-diphosphooligosaccharide--protein glycosyltransferase subunit 1	5	5	237.4	2.92E-03	2.1
Q6PD26	Pigs	GPI transamidase component PIG-S	2	2	101.2	1.32E-04	2.1
P68040	Rack1	Receptor of activated protein C kinase 1	3	3	143.9	5.88E-03	2.1
P58252	Eef2	Elongation factor 2	2	2	43.4	1.97E-03	2.1
Q01339	Apoh	Beta-2-glycoprotein 1	2	2	102.5	1.16E-05	2.0
P47758	Srprb	Signal recognition particle receptor subunit beta	2	2	88.2	4.38E-04	2.0
P62908	Rps3	40S ribosomal protein S3	2	2	118.0	7.18E-05	2.0
Q8VDN2	Atp1a1	Sodium/potassium-transporting ATPase subunit alpha-1	5	5	225.6	3.96E-05	2.0
Q8VEE1	Lmcd1	LIM and cysteine-rich domains protein 1	2	2	68.6	1.60E-05	2.0
G5E829	Atp2b1	Plasma membrane calcium-transporting ATPase 1	3	3	148.5	5.71E-04	1.9
Q9Z2I9	Sucla2	Succinate--CoA ligase [ADP-forming] subunit beta, mitochondrial	2	2	93.9	3.30E-03	1.9
P68368	Tuba4a	Tubulin alpha-4A chain	3	3	99.3	3.01E-03	1.9
P09470	Ace	Angiotensin-converting enzyme	3	3	54.4	2.47E-04	1.8
A2AUC9	Klhl41	Kelch-like protein 41	3	3	45.5	2.74E-04	1.7
Q9ERI6	Rdh14	Retinol dehydrogenase 14	2	2	95.9	7.16E-05	1.6

**Table 2:** Mass spectrometric identification of proteins identified by a single unique peptide with a reduced abundance in the sarcolemma-enriched fraction from dystrophic *mdx-4cv* skeletal muscle.

**Accession**	**Gene name**	**Protein name**	**Unique peptides**	**Peptide count**	**Confidence score**	**Anova (p)**	**Max fold change**
Q8R4V2	Dusp15	Dual specificity protein phosphatase 15	1	1	2.2	1.81E-11	Infinity
Q9D6F9	Tubb4a	Tubulin beta-4A chain	1	1	40.6	6.84E-04	Infinity
P45376	Akr1b1	Aldose reductase	1	1	2.3	1.60E-03	180.7
O08532	Cacna2d1	Voltage-dependent calcium channel subunit alpha-2/delta-1	1	1	46.7	7.89E-04	129.7
Q8BI84	Mia3	Melanoma inhibitory activity protein 3	1	1	2.6	6.58E-03	127.6
P17751	Tpi1	Triosephosphate isomerase	1	1	62.4	4.66E-03	80.4
Q6P8J7	Ckmt2	Creatine kinase S-type, mitochondrial	1	1	41.4	7.16E-04	53.0
Q9DB77	Uqcrc2	Cytochrome b-c1 complex subunit 2, mitochondrial	1	1	41.8	3.16E-03	49.4
P97384	Anxa11	Annexin A11	1	1	1.6	2.13E-03	44.4
P20917	Mag	Myelin-associated glycoprotein	1	1	2.6	2.60E-03	33.5
Q91VC4	Plvap	Plasmalemma vesicle-associated protein	1	1	49.0	7.80E-03	29.5
P05213	Tuba1b	Tubulin alpha-1B chain	1	1	1.6	1.08E-03	24.8
Q9CWZ7	Napg	Gamma-soluble NSF attachment protein	1	1	1.9	2.39E-03	24.7
Q9ERD7	Tubb3	Tubulin beta-3 chain	1	1	47.6	9.24E-05	18.0
P97450	Atp5j	ATP synthase-coupling factor 6, mitochondrial	1	1	5.9	8.57E-04	17.2
P04370	Mbp	Myelin basic protein	1	1	3.2	8.86E-05	16.8
O08749	Dld	Dihydrolipoyl dehydrogenase, mitochondrial	1	1	1.6	4.46E-03	12.2
Q3V1D3	Ampd1	AMP deaminase 1	1	1	56.3	4.51E-03	11.6
P17183	Eno2	Gamma-enolase	1	1	1.7	6.35E-03	8.4
Q8BMF4	Dlat	Dihydrolipoyllysine-residue acetyltransferase component of pyruvate dehydrogenase complex, mitochondrial	1	1	1.8	1.82E-03	8.4
P60202	Plp1	Myelin proteolipid protein	1	1	57.5	1.19E-05	8.3
P82349	Sgcb	Beta-sarcoglycan	1	1	41.5	7.17E-05	7.6
Q9CR68	Uqcrfs1	Cytochrome b-c1 complex subunit Rieske, mitochondrial	1	1	2.6	4.23E-04	7.4
Q9CWS0	Ddah1	N(G),N(G)-dimethylarginine dimethylaminohydrolase 1	1	1	57.3	1.68E-03	6.8
Q62425	Ndufa4	Cytochrome c oxidase subunit NDUFA4	1	1	1.8	6.61E-03	6.7
Q8BTJ4	Enpp4	Bis(5'-adenosyl)-triphosphatase enpp4	1	1	2.6	3.03E-04	6.3
Q6GQT9	Nomo1	Nodal modulator 1	1	1	2.0	4.13E-04	6.0
Q80XN0	Bdh1	D-beta-hydroxybutyrate dehydrogenase, mitochondrial	1	1	56.4	1.40E-03	6.0
Q9R0P9	Uchl1	Ubiquitin carboxyl-terminal hydrolase isozyme L1	1	1	1.9	1.24E-03	5.8
P70695	Fbp2	Fructose-1,6-bisphosphatase isozyme 2	1	1	55.8	3.66E-05	5.4
Q8CI12	Smtnl2	Smoothelin-like protein 2	1	1	3.3	7.12E-03	5.0
P20108	Prdx3	Thioredoxin-dependent peroxide reductase, mitochondrial	1	1	41.9	7.82E-06	4.8
Q1XH17	Trim72	Tripartite motif-containing protein 72	1	1	1.7	8.77E-03	4.8
O88456	Capns1	Calpain small subunit 1	1	1	2.7	3.57E-04	4.1
Q99LC5	Etfa	Electron transfer flavoprotein subunit alpha, mitochondrial	1	1	75.5	3.62E-03	3.9
Q9EQ20	Aldh6a1	Methylmalonate-semialdehyde dehydrogenase [acylating], mitochondrial	1	1	1.8	6.65E-03	3.6
P63038	Hspd1	60 kDa heat shock protein, mitochondrial	1	1	2.1	6.72E-04	3.6
P15532	Nme1	Nucleoside diphosphate kinase A	1	1	50.7	7.89E-03	3.6
Q17ST2	Kcna7	Potassium voltage-gated channel subfamily A member 7	1	1	1.7	2.01E-04	3.2
Q9CQR4	Acot13	Acyl-coenzyme A thioesterase 13	1	1	53.0	7.37E-04	3.0
P51174	Acadl	Long-chain specific acyl-CoA dehydrogenase, mitochondrial	1	1	1.8	6.40E-03	2.9
P12382	Pfkl	ATP-dependent 6-phosphofructokinase, liver type	1	1	2.0	1.54E-05	2.9
P06745	Gpi	Glucose-6-phosphate isomerase	1	1	1.6	7.64E-03	2.9
P21550	Eno3	Beta-enolase	1	1	51.8	8.45E-04	2.7
Q9D8B7	Jam3	Junctional adhesion molecule C	1	1	53.3	6.68E-03	2.6
P00015	Cyct	Cytochrome c, testis-specific	1	1	43.1	9.50E-04	2.5
P62077	Timm8b	Mitochondrial import inner membrane translocase subunit Tim8 B	1	1	2.1	3.27E-03	2.5
P56480	Atp5b	ATP synthase subunit beta, mitochondrial	1	1	69.4	2.02E-03	2.5
P35486	Pdha1	Pyruvate dehydrogenase E1 component subunit alpha, somatic form, mitochondrial	1	1	70.6	1.89E-03	2.5
P53395	Dbt	Lipoamide acyltransferase component of branched-chain alpha-keto acid dehydrogenase complex, mitochondrial	1	1	69.0	3.34E-04	2.4
Q64105	Spr	Sepiapterin reductase	1	1	51.0	1.18E-03	2.4
P10493	Nid1	Nidogen-1	1	1	45.8	5.02E-04	2.3
Q9D0F9	Pgm1	Phosphoglucomutase-1	1	1	55.2	7.26E-05	2.3
Q9R062	Gyg1	Glycogenin-1	1	1	56.9	6.08E-03	2.2
P31001	Des	Desmin	1	1	69.9	3.32E-04	2.1
Q9CZ13	Uqcrc1	Cytochrome b-c1 complex subunit 1, mitochondrial	1	1	2.7	2.28E-04	2.0
Q99MN9	Pccb	Propionyl-CoA carboxylase beta chain, mitochondrial	1	1	2.6	4.71E-03	2.0
Q8CI51	Pdlim5	PDZ and LIM domain protein 5	1	1	48.0	2.39E-03	2.0
Q62433	Ndrg1	Protein NDRG1	1	1	68.2	7.57E-03	1.9
P16125	Ldhb	L-lactate dehydrogenase B chain	1	1	51.2	1.59E-03	1.8
Q60714	Slc27a1	Long-chain fatty acid transport protein 1	1	1	2.4	9.45E-03	1.7
P63028	Tpt1	Translationally-controlled tumor protein	1	1	44.7	9.69E-03	1.6
Q99M71	Epdr1	Mammalian ependymin-related protein 1	1	1	89.1	9.58E-04	1.6
P62192	Psmc1	26S protease regulatory subunit 4	1	1	75.4	2.08E-03	1.5

**Table 3:** Mass spectrometric identification of proteins identified by a single unique peptide with an increased abundance in the sarcolemma-enriched fraction from dystrophic *mdx-4cv* skeletal muscle.

**Accession**	**Gene name**	**Protein name**	**Unique peptides**	**Peptide count**	**Confidence score**	**Anova (p)**	**Max fold change**
Q8BRK9	Man2a2	Alpha-mannosidase 2x	1	1	43.5	5.95E-04	Infinity
Q8R010	Aimp2	Aminoacyl tRNA synthase complex-interacting multifunctional protein 2	1	1	1.8	5.45E-04	Infinity
Q61009	Scarb1	Scavenger receptor class B member 1	1	1	53.7	9.03E-05	310.4
P16110	Lgals3	Galectin-3	1	1	49.6	3.94E-03	146.5
Q3U2S8	Hvcn1	Voltage-gated hydrogen channel 1	1	1	57.3	4.53E-05	115.8
Q62192	Cd180	CD180 antigen	1	1	1.9	7.90E-04	107.3
Q9CXJ4	Abcb8	ATP-binding cassette sub-family B member 8, mitochondrial	1	1	40.2	4.94E-05	101.6
P70402	Mybph	Myosin-binding protein H	1	1	3.9	1.16E-03	96.8
P62806	Hist1h4a	Histone H4	1	1	65.5	6.48E-03	65.7
P62911	Rpl32	60S ribosomal protein L32	1	1	2.4	2.30E-03	37.6
P41317	Mbl2	Mannose-binding protein C	1	1	53.9	1.08E-06	36.5
P43275	Hist1h1a	Histone H1.1	1	1	53.1	1.41E-06	31.7
P62717	Rpl18a	60S ribosomal protein L18a	1	1	41.5	8.72E-03	29.5
Q9DC16	Ergic1	Endoplasmic reticulum-Golgi intermediate compartment protein 1	1	1	1.8	1.99E-03	23.5
Q61093	Cybb	Cytochrome b-245 heavy chain	1	1	47.2	1.64E-03	22.8
O89017	Lgmn	Legumain	1	1	2.8	8.07E-03	20.7
Q9JM76	Arpc3	Actin-related protein 2/3 complex subunit 3	1	1	65.1	5.09E-04	15.6
P61514	Rpl37a	60S ribosomal protein L37a	1	1	55.2	7.44E-04	14.4
P84099	Rpl19	60S ribosomal protein L19	1	1	49.2	3.20E-06	14.0
P05555	Itgam	Integrin alpha-M	1	1	55.0	2.25E-05	14.0
P58771	Tpm1	Tropomyosin alpha-1 chain	1	1	61.2	9.47E-04	11.9
Q91V61	Sfxn3	Sideroflexin-3	1	1	41.2	7.69E-04	11.7
P11276	Fn1	Fibronectin	1	1	53.5	2.27E-07	11.5
P01843	1 SV	Ig lambda-1 chain C region	1	1	1.8	7.63E-05	11.1
Q9D3P8	Plgrkt	Plasminogen receptor (KT)	1	1	56.6	5.39E-04	8.7
P13542	Myh8	Myosin-8	1	1	53.5	3.30E-03	8.5
Q8BTM8	Flna	Filamin-A	1	1	1.6	6.18E-03	8.4
Q99P91	Gpnmb	Transmembrane glycoprotein NMB	1	1	3.3	1.91E-05	8.2
P10126	Eef1a1	Elongation factor 1-alpha 1	1	1	1.8	2.27E-05	8.0
P30355	Alox5ap	Arachidonate 5-lipoxygenase-activating protein	1	1	44.6	6.95E-06	7.7
Q9Z1G4	Atp6v0a1	V-type proton ATPase 116 kDa subunit a isoform 1	1	1	43.3	3.90E-04	7.6
P63158	Hmgb1	High mobility group protein B1	1	1	62.0	3.60E-03	7.6
P03911	Mtnd4	NADH-ubiquinone oxidoreductase chain 4	1	1	45.2	1.13E-03	7.4
P11688	Itga5	Integrin alpha-5	1	1	2.2	2.69E-03	7.2
P62855	Rps26	40S ribosomal protein S26	1	1	1.9	6.29E-06	7.2
Q64735	Cr1l	Complement component receptor 1-like protein	1	1	1.8	1.82E-04	7.1
P62849	Rps24	40S ribosomal protein S24	1	1	1.8	5.43E-06	6.9
P61211	Arl1	ADP-ribosylation factor-like protein 1	1	1	64.9	1.53E-03	6.6
P01631	1 SV	Ig kappa chain V-II region 26-10	1	1	1.7	2.89E-03	6.5
Q8BP67	Rpl24	60S ribosomal protein L24	1	1	62.0	5.82E-04	6.5
Q3THE2	Myl12b	Myosin regulatory light chain 12B	1	1	58.3	1.73E-04	6.4
P62830	Rpl23	60S ribosomal protein L23	1	1	44.3	7.44E-06	6.4
Q9Z0M6	Cd97	CD97 antigen	1	1	59.5	3.19E-04	6.3
P14869	Rplp0	60S acidic ribosomal protein P0	1	1	56.5	2.55E-04	6.3
Q80VQ0	Aldh3b1	Aldehyde dehydrogenase family 3 member B1	1	1	63.4	3.55E-06	6.0
P14429	H2-Q7	H-2 class I histocompatibility antigen, Q7 alpha chain	1	1	53.0	1.09E-03	6.0
Q8K396	Mnd1	Meiotic nuclear division protein 1 homolog	1	1	41.5	3.34E-03	5.7
P62892	Rpl39	60S ribosomal protein L39	1	1	2.7	2.37E-04	5.7
P17426	Ap2a1	AP-2 complex subunit alpha-1	1	1	46.5	3.94E-03	5.7
Q61704	Itih3	Inter-alpha-trypsin inhibitor heavy chain H3	1	1	1.8	4.27E-05	5.6
Q8VCM7	Fgg	Fibrinogen gamma chain	1	1	2.3	3.23E-06	5.6
P13412	Tnni2	Troponin I, fast skeletal muscle	1	1	58.2	9.73E-03	5.5
Q9Z1D1	Eif3g	Eukaryotic translation initiation factor 3 subunit G	1	1	1.8	3.69E-03	5.5
Q62188	Dpysl3	Dihydropyrimidinase-related protein 3	1	1	2.3	5.61E-04	5.4
P53702	Hccs	Cytochrome c-type heme lyase	1	1	1.8	8.06E-03	5.4
P14483	H2-Ab1	H-2 class II histocompatibility antigen, A beta chain	1	1	44.8	3.42E-03	5.4
P07356	Anxa2	Annexin A2	1	1	49.0	3.65E-06	5.3
P14434	H2-Aa	H-2 class II histocompatibility antigen, A-B alpha chain	1	1	47.0	1.75E-09	5.3
Q8K449	Abca9	ATP-binding cassette sub-family A member 9	1	1	1.6	1.36E-03	5.3
Q3V3R4	Itga1	Integrin alpha-1	1	1	67.3	6.35E-04	5.2
Q9CXW4	Rpl11	60S ribosomal protein L11	1	1	49.0	3.90E-03	5.2
P55772	Entpd1	Ectonucleoside triphosphate diphosphohydrolase 1	1	1	43.0	3.07E-05	5.2
Q8BK08	Tmem11	Transmembrane protein 11, mitochondrial	1	1	2.1	5.27E-05	5.0
Q61102	Abcb7	ATP-binding cassette sub-family B member 7, mitochondrial	1	1	2.4	1.77E-03	5.0
P62962	Pfn1	Profilin-1	1	1	42.6	2.40E-03	5.0
P03899	Mtnd3	NADH-ubiquinone oxidoreductase chain 3	1	1	47.8	1.08E-03	5.0
Q9Z2G6	Sel1l	Protein sel-1 homolog 1	1	1	2.5	8.61E-04	4.9
P60335	Pcbp1	Poly(rC)-binding protein 1	1	1	55.0	1.55E-04	4.9
Q06890	Clu	Clusterin	1	1	57.2	6.12E-06	4.9
Q9JK37	Myoz1	Myozenin-1	1	1	88.8	1.86E-03	4.7
Q91VC9	Ghitm	Growth hormone-inducible transmembrane protein	1	1	1.9	4.82E-06	4.7
Q8BUY5	Timmdc1	Complex I assembly factor TIMMDC1, mitochondrial	1	1	1.5	5.83E-04	4.7
Q60930	Vdac2	Voltage-dependent anion-selective channel protein 2	1	1	60.0	2.49E-05	4.6
Q9D5T0	Atad1	ATPase family AAA domain-containing protein 1	1	1	41.7	8.45E-04	4.6
Q9D783	Klhl40	Kelch-like protein 40	1	1	55.1	4.21E-05	4.6
Q9CZU6	Cs	Citrate synthase, mitochondrial	1	1	50.2	5.75E-03	4.6
Q61233	Lcp1	Plastin-2	1	1	1.7	2.25E-04	4.6
Q78IK2	Usmg5	Up-regulated during skeletal muscle growth protein 5	1	1	40.3	6.83E-03	4.5
P35564	Canx	Calnexin	1	1	52.9	3.89E-05	4.5
P01869	Ighg1	Ig gamma-1 chain C region, membrane-bound form	1	1	2.3	3.82E-04	4.4
Q60605	Myl6	Myosin light polypeptide 6	1	1	74.6	9.77E-05	4.4
Q9D1G3	Hhatl	Protein-cysteine N-palmitoyltransferase HHAT-like protein	1	1	1.8	4.25E-06	4.4
P01867	Igh-3	Ig gamma-2B chain C region	1	1	46.8	3.25E-03	4.4
Q3TMP8	Tmem38a	Trimeric intracellular cation channel type A	1	1	48.3	6.99E-03	4.3
Q7TSH2	Phkb	Phosphorylase b kinase regulatory subunit beta	1	1	47.6	1.27E-04	4.3
Q9D1L0	Chchd2	Coiled-coil-helix-coiled-coil-helix domain-containing protein 2	1	1	41.2	8.88E-08	4.2
P61255	Rpl26	60S ribosomal protein L26	1	1	1.5	5.18E-05	4.2
Q9R0X4	Acot9	Acyl-coenzyme A thioesterase 9, mitochondrial	1	1	43.4	7.66E-03	4.2
Q62351	Tfrc	Transferrin receptor protein 1	1	1	1.7	7.22E-04	4.2
Q8BJU2	Tspan9	Tetraspanin-9	1	1	65.0	2.22E-03	4.2
P07091	S100a4	Protein S100-A4	1	1	1.5	1.73E-05	4.1
Q922Q8	Lrrc59	Leucine-rich repeat-containing protein 59	1	1	42.3	1.66E-04	4.1
O54724	Ptrf	Polymerase I and transcript release factor	1	1	96.2	9.33E-03	4.1
A2AMM0	Murc	Muscle-related coiled-coil protein	1	1	44.4	1.31E-03	4.1
P62751	Rpl23a	60S ribosomal protein L23a	1	1	1.7	2.69E-05	4.1
O88587	Comt	Catechol O-methyltransferase	1	1	45.0	1.83E-03	4.0
Q9D1E8	Agpat5	1-acyl-sn-glycerol-3-phosphate acyltransferase epsilon	1	1	75.1	3.34E-04	4.0
P57716	Ncstn	Nicastrin	1	1	2.5	3.11E-03	4.0
Q91WC3	Acsl6	Long-chain-fatty-acid--CoA ligase 6	1	1	45.0	3.20E-03	3.9
P14685	Psmd3	26S proteasome non-ATPase regulatory subunit 3	1	1	2.0	4.17E-04	3.9
Q8BU33	Ilvbl	Acetolactate synthase-like protein	1	1	1.6	3.98E-04	3.8
Q5U458	Dnajc11	DnaJ homolog subfamily C member 11	1	1	2.5	1.60E-04	3.8
Q8VIB3	St3gal6	Type 2 lactosamine alpha-2,3-sialyltransferase	1	1	2.0	1.73E-03	3.8
P01864	1 SV	Ig gamma-2A chain C region secreted form	1	1	53.3	7.22E-04	3.8
Q9Z2A9	Ggt5	Gamma-glutamyltransferase 5	1	1	2.7	2.86E-04	3.8
Q91WS0	Cisd1	CDGSH iron-sulfur domain-containing protein 1	1	1	2.2	9.61E-04	3.7
P17182	Eno1	Alpha-enolase	1	1	1.9	3.48E-03	3.7
Q9D8B4	Ndufa11	NADH dehydrogenase [ubiquinone] 1 alpha subcomplex subunit 11	1	1	44.0	6.74E-04	3.7
P43277	Hist1h1d	Histone H1.3	1	1	2.4	1.78E-05	3.6
Q8VHW3	Cacng6	Voltage-dependent calcium channel gamma-6 subunit	1	1	43.2	5.85E-03	3.5
Q8BH61	F13a1	Coagulation factor XIII A chain	1	1	49.3	1.98E-04	3.5
Q8BTX9	Hsdl1	Inactive hydroxysteroid dehydrogenase-like protein 1	1	1	40.7	2.16E-03	3.5
P60710	Actb	Actin, cytoplasmic 1	1	1	52.6	4.65E-03	3.5
P10922	H1f0	Histone H1.0	1	1	1.7	3.98E-03	3.5
P16406	Enpep	Glutamyl aminopeptidase	1	1	2.1	2.51E-05	3.4
P24668	M6pr	Cation-dependent mannose-6-phosphate receptor	1	1	48.8	4.15E-04	3.4
Q9JLH8	Tmod4	Tropomodulin-4	1	1	76.8	7.69E-03	3.4
Q9QXS1	Plec	Plectin	1	1	61.5	1.19E-04	3.3
Q80UU9	Pgrmc2	Membrane-associated progesterone receptor component 2	1	1	1.9	1.11E-05	3.3
Q925F2	Esam	Endothelial cell-selective adhesion molecule	1	1	51.9	4.23E-05	3.2
Q9JJI8	Rpl38	60S ribosomal protein L38	1	1	89.2	3.00E-03	3.2
Q8BHC4	Dcakd	Dephospho-CoA kinase domain-containing protein	1	1	2.1	5.45E-03	3.1
P15508	Sptb	Spectrin beta chain, erythrocytic	1	1	45.8	3.12E-03	3.1
Q9DBG6	Rpn2	Dolichyl-diphosphooligosaccharide--protein glycosyltransferase subunit 2	1	1	67.1	9.56E-03	3.1
Q8BJS4	Sun2	SUN domain-containing protein 2	1	1	2.1	1.85E-05	3.1
Q3TDQ1	Stt3b	Dolichyl-diphosphooligosaccharide--protein glycosyltransferase subunit STT3B	1	1	2.0	3.65E-03	3.0
P62835	Rap1a	Ras-related protein Rap-1A	1	1	46.0	1.42E-03	3.0
Q8BT35	4 SV	Uncharacterized protein LINC00116 homolog	1	1	48.4	1.11E-05	3.0
P51912	Slc1a5	Neutral amino acid transporter B(0)	1	1	61.7	3.15E-05	3.0
Q3TL44	Nlrx1	NLR family member X1	1	1	58.5	8.72E-05	2.9
O08529	Capn2	Calpain-2 catalytic subunit	1	1	53.8	6.94E-03	2.9
Q9ESD7	Dysf	Dysferlin	1	1	47.5	2.84E-03	2.8
Q8VCL2	Sco2	Protein SCO2 homolog, mitochondrial	1	1	72.7	6.29E-03	2.8
Q8R429	Atp2a1	Sarcoplasmic/endoplasmic reticulum calcium ATPase 1	1	1	1.9	3.46E-03	2.8
O70209	Pdlim3	PDZ and LIM domain protein 3	1	1	49.7	2.03E-04	2.7
O88342	Wdr1	WD repeat-containing protein 1	1	1	40.9	6.82E-03	2.7
Q9Z1T2	Thbs4	Thrombospondin-4	1	1	1.7	8.87E-03	2.7
Q62234	Myom1	Myomesin-1	1	1	2.0	1.43E-03	2.7
Q64310	Surf4	Surfeit locus protein 4	1	1	52.8	2.58E-04	2.7
Q08481	Pecam1	Platelet endothelial cell adhesion molecule	1	1	1.6	1.17E-03	2.7
Q00623	Apoa1	Apolipoprotein A-I	1	1	41.7	2.62E-03	2.7
P35330	Icam2	Intercellular adhesion molecule 2	1	1	47.3	2.08E-04	2.7
Q921T2	Tor1aip1	Torsin-1A-interacting protein 1	1	1	1.9	1.50E-03	2.6
P18760	Cfl1	Cofilin-1	1	1	2.1	4.91E-03	2.6
Q91YT0	Ndufv1	NADH dehydrogenase [ubiquinone] flavoprotein 1, mitochondrial	1	1	50.8	2.26E-03	2.6
P62821	Rab1A	Ras-related protein Rab-1A	1	1	48.3	2.70E-04	2.6
P01831	Thy1	Thy-1 membrane glycoprotein	1	1	79.3	3.29E-03	2.6
P27601	Gna13	Guanine nucleotide-binding protein subunit alpha-13	1	1	69.3	7.00E-03	2.6
Q9CRD0	Ociad1	OCIA domain-containing protein 1	1	1	2.1	1.04E-04	2.6
P14733	Lmnb1	Lamin-B1	1	1	50.9	8.80E-03	2.5
Q61207	Psap	Prosaposin	1	1	82.5	1.58E-03	2.5
O35604	Npc1	Niemann-Pick C1 protein	1	1	43.1	3.63E-03	2.5
P23927	Cryab	Alpha-crystallin B chain	1	1	42.7	9.38E-03	2.5
P01027	C3	Complement C3	1	1	1.9	1.79E-03	2.5
Q9CZW5	Tomm70	Mitochondrial import receptor subunit TOM70	1	1	49.4	5.99E-03	2.5
Q7TQ95	Lnp	Protein lunapark	1	1	61.7	1.55E-04	2.5
Q6ZWY3	Rps27l	40S ribosomal protein S27-like	1	1	91.0	4.64E-05	2.5
Q6PCP5	Mff	Mitochondrial fission factor	1	1	2.2	9.63E-03	2.5
Q9CZX8	Rps19	40S ribosomal protein S19	1	1	49.3	6.83E-04	2.5
P97443	Smyd1	Histone-lysine N-methyltransferase Smyd1	1	1	1.6	8.33E-04	2.5
P51150	Rab7a	Ras-related protein Rab-7a	1	1	53.4	4.97E-03	2.4
O55222	Ilk	Integrin-linked protein kinase	1	1	66.1	7.29E-03	2.4
O70503	Hsd17b12	Very-long-chain 3-oxoacyl-CoA reductase	1	1	2.0	2.05E-04	2.4
P50543	S100a11	Protein S100-A11	1	1	51.4	7.19E-03	2.4
Q8BGH2	Samm50	Sorting and assembly machinery component 50 homolog	1	1	63.8	6.89E-03	2.4
Q3TC33	Ccdc127	Coiled-coil domain-containing protein 127	1	1	1.7	2.30E-03	2.4
Q9CR62	Slc25a11	Mitochondrial 2-oxoglutarate/malate carrier protein	1	1	41.0	9.42E-04	2.4
Q9Z0X1	Aifm1	Apoptosis-inducing factor 1, mitochondrial	1	1	2.5	3.64E-03	2.3
Q8BH64	Ehd2	EH domain-containing protein 2	1	1	1.9	4.58E-04	2.3
Q8JZN7	Rhot2	Mitochondrial Rho GTPase 2	1	1	2.2	6.43E-04	2.3
Q9DC70	Ndufs7	NADH dehydrogenase [ubiquinone] iron-sulfur protein 7, mitochondrial	1	1	50.9	2.12E-03	2.3
O70423	Aoc3	Membrane primary amine oxidase	1	1	69.0	4.91E-03	2.3
Q8BXZ1	Tmx3	Protein disulfide-isomerase TMX3	1	1	48.6	8.14E-05	2.3
Q3V009	Tmed1	Transmembrane emp24 domain-containing protein 1	1	1	59.2	1.70E-03	2.2
P50516	Atp6v1a	V-type proton ATPase catalytic subunit A	1	1	42.8	1.49E-05	2.2
Q9DAS9	Gng12	Guanine nucleotide-binding protein G(I)/G(S)/G(O) subunit gamma-12	1	1	43.8	9.49E-04	2.2
P61222	Abce1	ATP-binding cassette sub-family E member 1	1	1	42.1	2.93E-03	2.2
Q8R404	Mic13	MIC complex subunit MIC13	1	1	2.0	7.14E-03	2.2
P10833	Rras	Ras-related protein R-Ras	1	1	61.8	4.00E-03	2.1
Q9R069	Bcam	Basal cell adhesion molecule	1	1	63.1	7.93E-03	2.1
P08752	Gnai2	Guanine nucleotide-binding protein G(i) subunit alpha-2	1	1	45.6	8.27E-03	2.1
Q9ET78	Jph2	Junctophilin-2	1	1	40.4	3.14E-03	2.1
Q99LR1	Abhd12	Monoacylglycerol lipase ABHD12	1	1	1.7	2.20E-03	2.1
Q9D0F3	Lman1	Protein ERGIC-53	1	1	71.3	1.29E-04	2.1
Q9WTK3	Gpaa1	Glycosylphosphatidylinositol anchor attachment 1 protein	1	1	40.2	7.52E-03	2.1
P14231	Atp1b2	Sodium/potassium-transporting ATPase subunit beta-2	1	1	93.7	2.41E-04	2.0
Q60854	Serpinb6	Serpin B6	1	1	80.9	2.16E-04	2.0
Q78IS1	Tmed3	Transmembrane emp24 domain-containing protein 3	1	1	43.8	6.97E-04	2.0
O88983	Stx8	Syntaxin-8	1	1	144.0	3.98E-05	2.0
Q01149	Col1a2	Collagen alpha-2(I) chain	1	1	40.2	3.41E-03	2.0
Q5NBX1	Cobl	Protein cordon-bleu	1	1	40.8	5.32E-03	1.9
Q9JIF9	Myot	Myotilin	1	1	1.7	6.83E-04	1.9
Q9CQJ8	Ndufb9	NADH dehydrogenase [ubiquinone] 1 beta subcomplex subunit 9	1	1	2.1	3.94E-03	1.9
P61226	Rap2b	Ras-related protein Rap-2b	1	1	67.8	2.55E-03	1.9
P00329	Adh1	Alcohol dehydrogenase 1	1	1	1.5	6.12E-03	1.9
P47955	Rplp1	60S acidic ribosomal protein P1	1	1	3.1	1.05E-04	1.8
Q9DBL1	Acadsb	Short/branched chain specific acyl-CoA dehydrogenase, mitochondrial	1	1	93.0	6.83E-03	1.8
Q9CQY5	Magt1	Magnesium transporter protein 1	1	1	66.1	9.76E-03	1.8
Q9D6Y9	Gbe1	1,4-alpha-glucan-branching enzyme	1	1	1.7	4.16E-03	1.8
Q80W54	Zmpste24	CAAX prenyl protease 1 homolog	1	1	1.7	2.15E-03	1.8
P70452	Stx4	Syntaxin-4	1	1	71.6	3.04E-03	1.8
Q9CXD6	Mcur1	Mitochondrial calcium uniporter regulator 1	1	1	2.3	1.77E-03	1.8
P26041	Msn	Moesin	1	1	42.9	7.89E-03	1.8
Q791V5	Mtch2	Mitochondrial carrier homolog 2	1	1	1.6	1.99E-03	1.7
Q8JZN5	Acad9	Acyl-CoA dehydrogenase family member 9, mitochondrial	1	1	67.3	1.55E-03	1.7
Q8CHS7	Dhrs7c	Dehydrogenase/reductase SDR family member 7C	1	1	40.1	5.80E-03	1.7
Q76MZ3	Ppp2r1a	Serine/threonine-protein phosphatase 2A 65 kDa regulatory subunit A alpha isoform	1	1	62.3	5.92E-03	1.7
P14069	S100a6	Protein S100-A6	1	1	66.7	7.20E-03	1.7
P11499	Hsp90ab1	Heat shock protein HSP 90-beta	1	1	3.3	2.57E-03	1.7
Q61335	Bcap31	B-cell receptor-associated protein 31	1	1	2.0	1.22E-03	1.6
Q69ZQ1	Kiaa1161	Uncharacterized family 31 glucosidase KIAA1161	1	1	40.5	1.58E-03	1.6
P61028	Rab8b	Ras-related protein Rab-8B	1	1	41.8	4.44E-04	1.5
Q64727	Vcl	Vinculin	1	1	55.2	1.52E-03	1.5

## 2 Experimental design, materials and methods

Details of the methodological approach used in this study are available in [1].

### 2.1 Research animal population

Combined hind limb muscle specimens from dystrophic *mdx-4cv* mice and age-matched wild type mice (4 g wet weight; n = 3) were used for tissue extraction. For each biological replicate, three hind legs from three different mice were pooled. Thus, in total nine hind legs were used to generate three biological replicates each for wild type and *mdx-4cv* specimens. All procedures are described in the associated research article [1].

### 2.2 Subcellular fractionation and protein extraction

Using crude microsomal membranes as starting material, an enriched sarcolemma preparation was isolated from normal versus dystrophic mouse muscle employing an established lectin agglutination procedure, as previously described in detail [2], [3], [4]. De-agglutination was carried out by incubation with the competitive sugar 0.2 M N-acetyl-D-glucosamine [5]. All procedures are described in the associated research article [1].

### 2.3 Mass spectrometric analysis of muscle proteins

The concentration of sarcolemma protein samples was determined using the Bradford assay system [6] and sample volumes were equalized with label-free solubilization buffer. For LC-MS/MS analysis, samples were reduced with 10 mM dithiothreitol for 30 minutes at room temperature and then alkylated with 25 mM iodoacteamide in 50 mM ammonium bicarbonate for 20 minutes at room temperature in the dark [7]. The proteolytic digestion of normal versus dystrophic specimens was initially carried out with sequencing grade Lys-C at a ratio of 1:100 (protease:protein) at 37 °C for 4 hours. Following dilution of samples with four times the initial sample volume with 50 mM ammonium bicarbonate, a second digestion step was performed with sequencing grade modified trypsin at a ratio of 1:25 (protease:protein) overnight at 37 °C [8]. LC-MS/MS analysis was carried out using an Ultimate 3000 NanoLC system (Dionex Corporation, Sunnyvale, CA, USA) coupled to a Q-Exactive mass spectrometer (Thermo Fisher Scientific). The mass spectrometer was operated in positive, data-dependent mode and was externally calibrated. All analytical procedures are described in the associated research article [1].
